# Paraneoplastic maculopathy mimicking diabetic macular edema

**DOI:** 10.3205/oc000269

**Published:** 2026-04-16

**Authors:** Durgul Acan, Yurdagul Girgin, Eyyup Karahan

**Affiliations:** 1 Balıkesir University School of Medicine, Department of Ophthalmology, Balıkesir, Turkey; 2 Tekirdag Corlu State Hospital, Department of Ophthalmology, Tekirdağ, Turkey

**Keywords:** diabetic macular edema, optical coherence tomography, paraproteinemic maculopathy, serous retinal detachment

## Abstract

**Objective:**

To highlight paraproteinemic maculopathy as a potential initial manifestation of multiple myeloma and to emphasize its ability to mimic diabetic macular edema in patients with diabetes.

**Methods:**

Two diabetic patients presenting with macular edema unresponsive to intravitreal anti-vascular endothelial growth factor therapy were evaluated. Detailed ophthalmic examination and fundus fluorescein angiography (FFA) were performed, followed by systemic investigations.

**Results:**

Subsequent systemic evaluation led to the diagnosis of multiple myeloma in both patients and with systemic therapy, a marked regression of findings was observed in all eyes.

**Conclusions:**

Paraproteinemic maculopathy may present as an initial ocular manifestation of multiple myeloma and can closely mimic diabetic macular edema. It should be considered in diabetic patients with atypical macular findings, particularly therapy nonresponsive serous macular detachment.

## Introduction

Paraproteinemic maculopathy (PM) is characterized by serous retinal detachment (SRD) of the macula and frequently it arises from an elevation of a monoclonal antibody in the blood, associated with immunoproliferative diseases [1]. SRD in PM can resemble central serous chorioretinopathy, polypoidal choroidal vasculopathy, age-related macular degeneration, Harada’s disease, retinal macroaneurysm, and retinal vein occlusion [2]. In patients with diabetes, the localized SRD at the macula seen on optical coherence tomography (OCT) may lead to the misdiagnosis of diabetic macular edema (DME) and may result in unnecessary intravitreal treatments [3], [4]. In this report, two diabetic patients with maculopathy demonstrated suboptimal responses to intravitreal anti-vascular endothelial growth factor (anti-VEGF) therapy and were subsequently diagnosed with PM.

## Case descriptions

### Case 1

**Figure 1:**
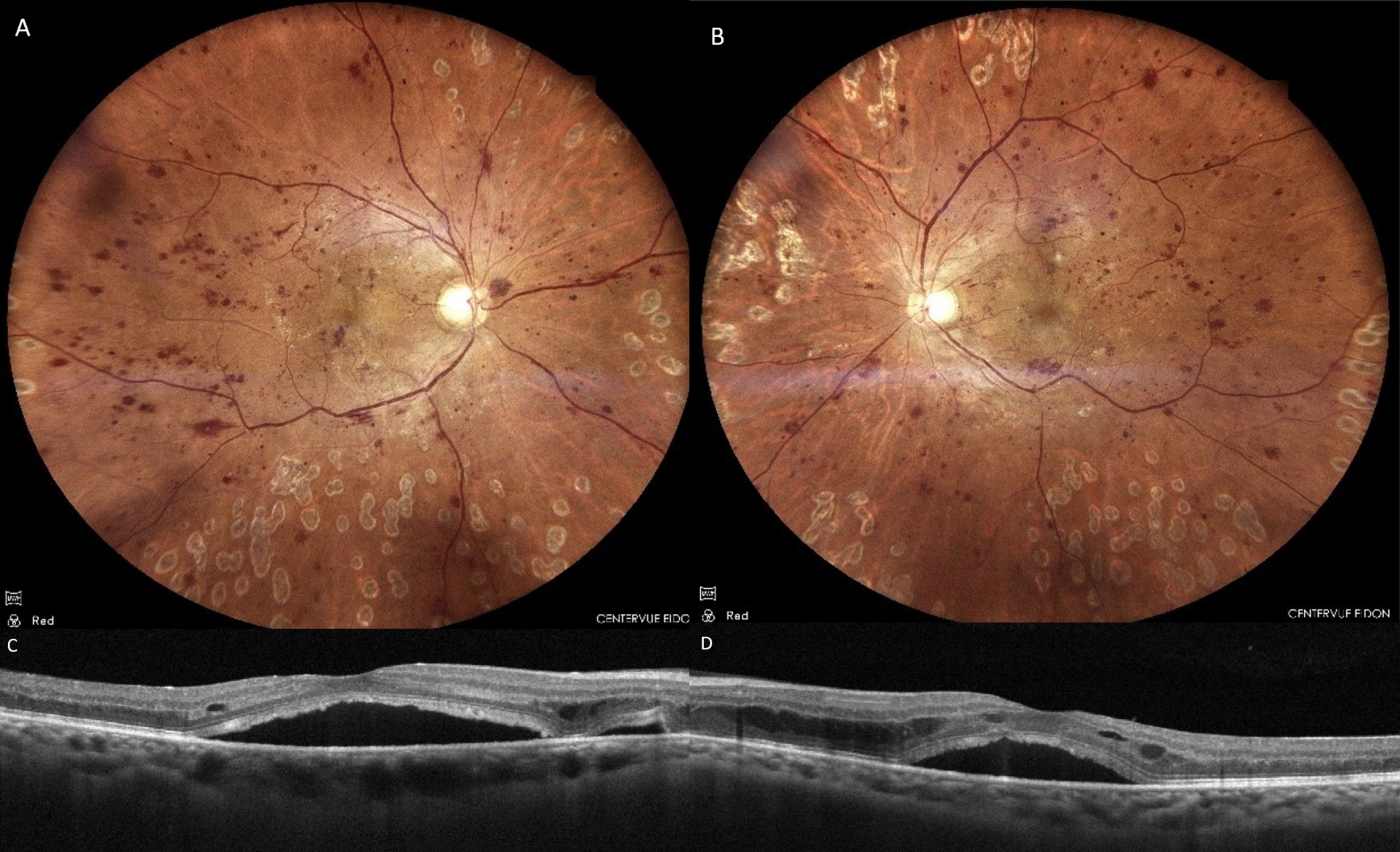
Case 1; color fundus and OCT images at initial presentation. Extensive retinal hemorrhages and microaneurysms with occasional laser spots are seen in right (A) and left (B) eyes. Significant subretinal fluid and intraretinal edema were also observed on simultaneous OCT images of the right (C) and the left (D) eyes.

A 52-year-old male patient with a 12-year history of diabetes presented to our department reporting progressive visual impairment in both eyes. He had no other known systemic diseases. On ophthalmological examination, the best-corrected visual acuity (BCVA) was 20/50 in the right eye and 20/30 in the left eye with Snellen charts, bilateral biomicroscopic evaluations of anterior segments were normal, and intraocular pressure (IOP) measurements were within normal range for both eyes. Dilated fundus examination revealed widespread hemorrhages and microaneurysms in all quadrants of both retinas and laser spots, consistent with diabetic retinopathy (DR) (Figure 1A and B (Fig. 1)). Bilateral SRD and intraretinal fluid accumulation, which was more prominent in the left eye, were detected on OCT (Figure 1C and D (Fig. 1)). The patient was diagnosed with DR and DME; intravitreal bevacizumab injections were advised.

**Figure 2:**
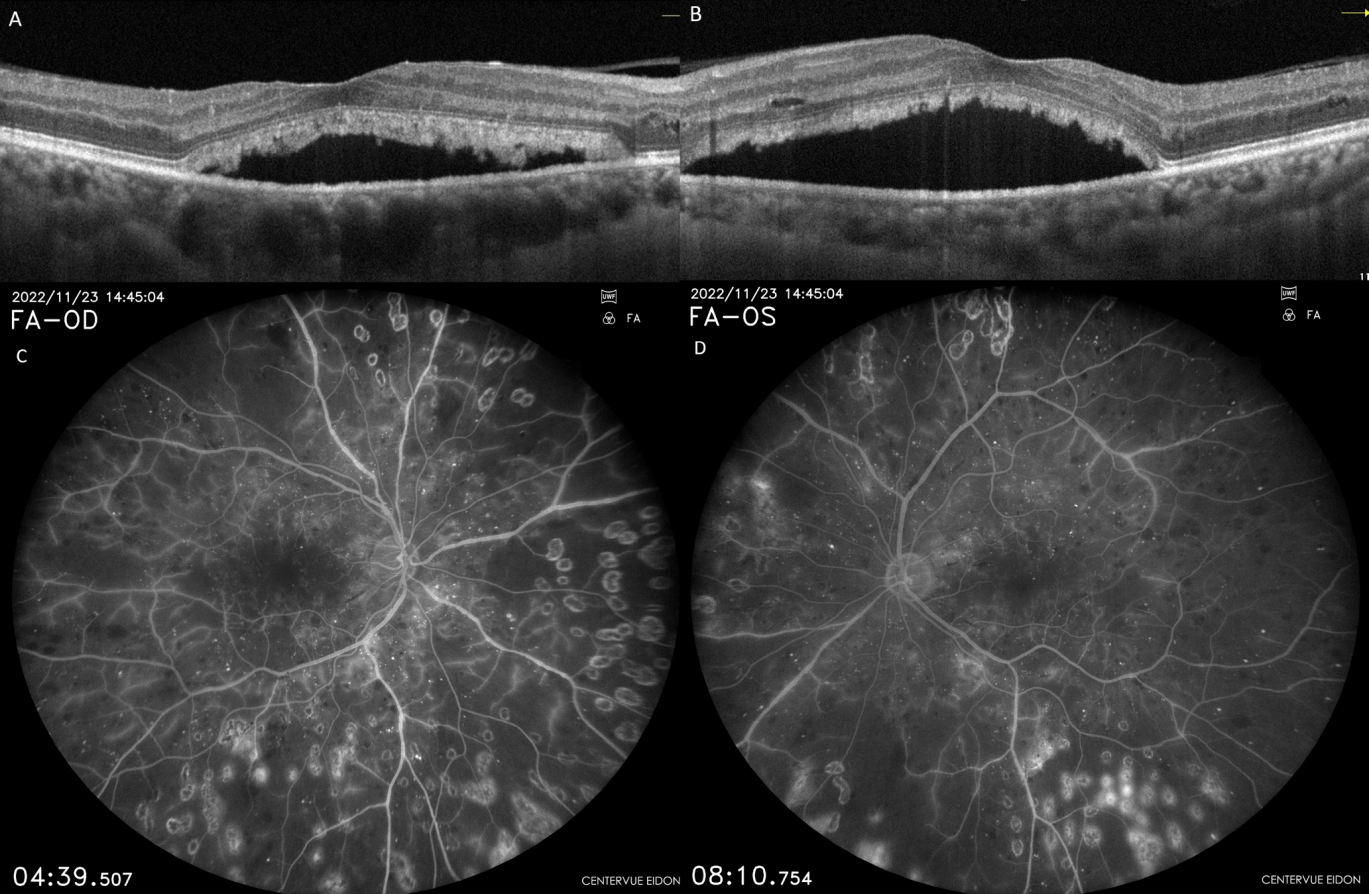
Case 1; OCT and FFA images of the right (A, C) and left (B, D) eyes after the anti-VEGF upload therapy. Intraretinal edema has regressed, but subretinal fluid persists and photoreceptor shedding becomes more apparent. FFA shows bilateral large retinal ischemia.

**Figure 3:**
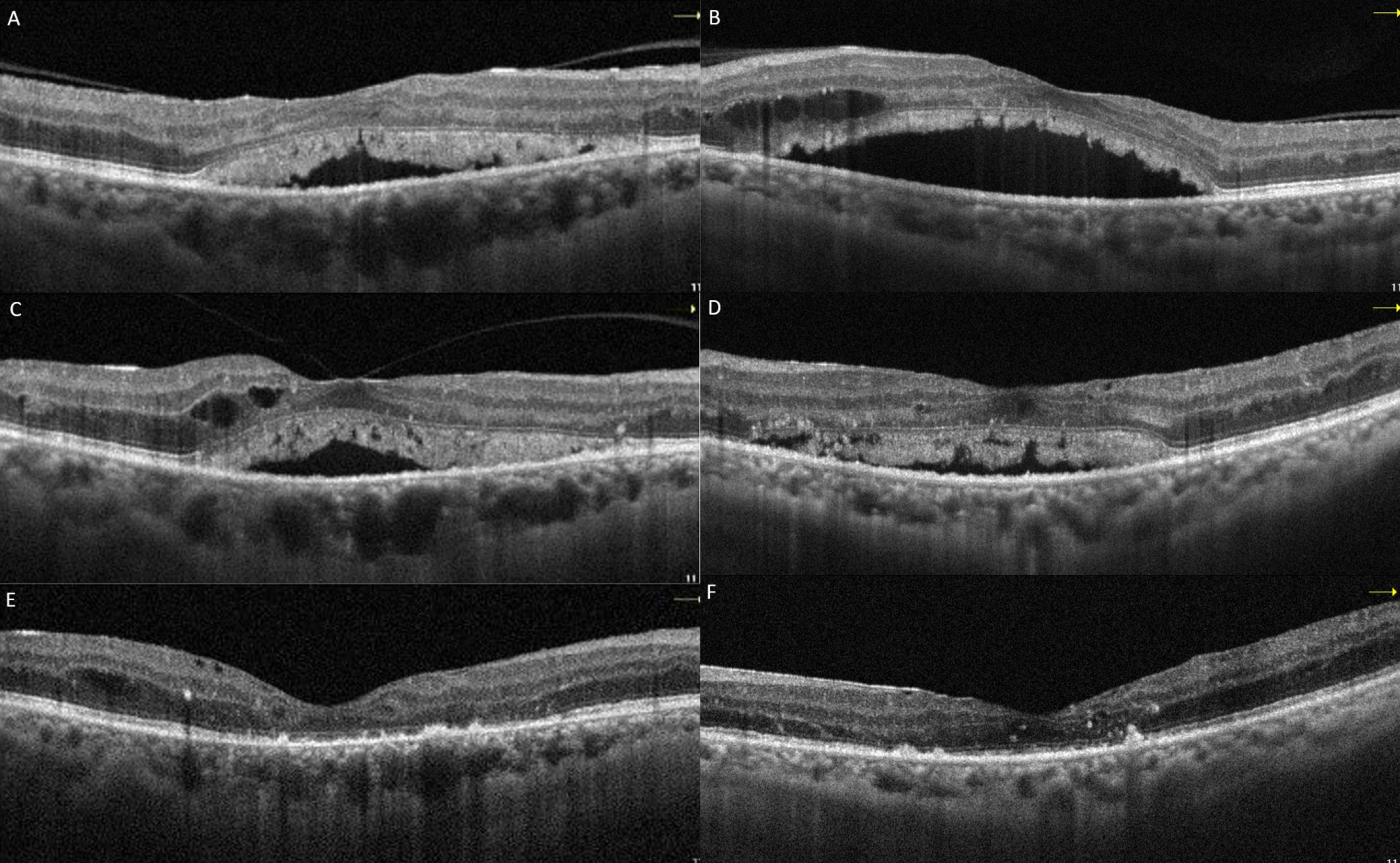
Macular OCT images of case 1 at 1 month (A: right, B: left), 3 months (C: right, D: left), and 1 year (E: right, F: left) after the initiation of systemic chemotherapy showed resolution of the subretinal fluid and shed photoreceptors.

Following three intravitreal bevacizumab injections administered to both eyes, the patient’s BCVA declined to 20/100 in the right eye and 20/60 in the left eye. OCT images showed a decrease in intraretinal fluid, but there was no regression in SRDs (Figure 2A and B (Fig. 2)). Continuing panretinal photocoagulation was scheduled due to retinal ischemia on fundus fluorescein angiography (FFA) (Figure 2C and D (Fig. 2)). However, since the persistence of the SRD, no apparent leakage at the macula on FFA (Figure 2C and D (Fig. 2)), photoreceptor shedding was initiated on OCT (Figure 2A and B (Fig. 2)), and the patient complained of increased weakness and fatigue, an internal medicine consultation was requested. Laboratory tests showed hemoglobulin 6.8 g/dl (normal range: 13.1–17.2 g/dl), albumin 23 g/l (normal range: 35–52 g/dl), erythrocyte sedimentation rate 140 mm/h (normal value: <15 mm/h), C-reactive protein 14.9 mg/l (normal value: <6 mg/l) and hemoglobin A1c 8.1% (normal range: 3.5–5.7%) in the blood sample, and there was +3 proteinuria in the urine. Lymphadenopathy was also detected in the left inguinal region. Abdominal computerized tomography revealed lytic bone lesions in the left iliac wing, right acetabulum and L3–L4 vertebral bodies. The patient was diagnosed with multiple myeloma (MM) based on protein electrophoresis and hematologic examination. He was subsequently started on systemic chemotherapy for MM and no additional injections were administered for macular edema. Following initiation of treatment, macular subretinal fluid gradually resolved bilaterally (Figure 3A–F (Fig. 3)). The final OCT examination, 1 year after the first presentation showed resolution of the serous macular detachment (Figure 3E and F (Fig. 3)). At that time BCVA was 20/80 in the right eye and 20/100 in the left eye.

### Case 2

A 68-year-old male patient was admitted with a complaint of vision loss in the left eye. He had an 8-year history of type 2 diabetes mellitus that was managed with oral antidiabetic agents and no other known systemic comorbidities. On ophthalmologic examination, BCVA was 20/25 in the right eye with Snellen charts and counting fingers at 50 cm on the left; the right eye was pseudophakic, and there was a mature cataract on the left. Dilated fundus examination revealed retinal hemorrhages and cystoid macular edema in the right eye, consistent with diabetic DR and DME. Fundus details in the left eye were undetectable due to the dense cataract, but ultrasonographically, the retina was attached and the vitreous was clear. Phacoemulsification surgery was initially planned.

**Figure 4:**
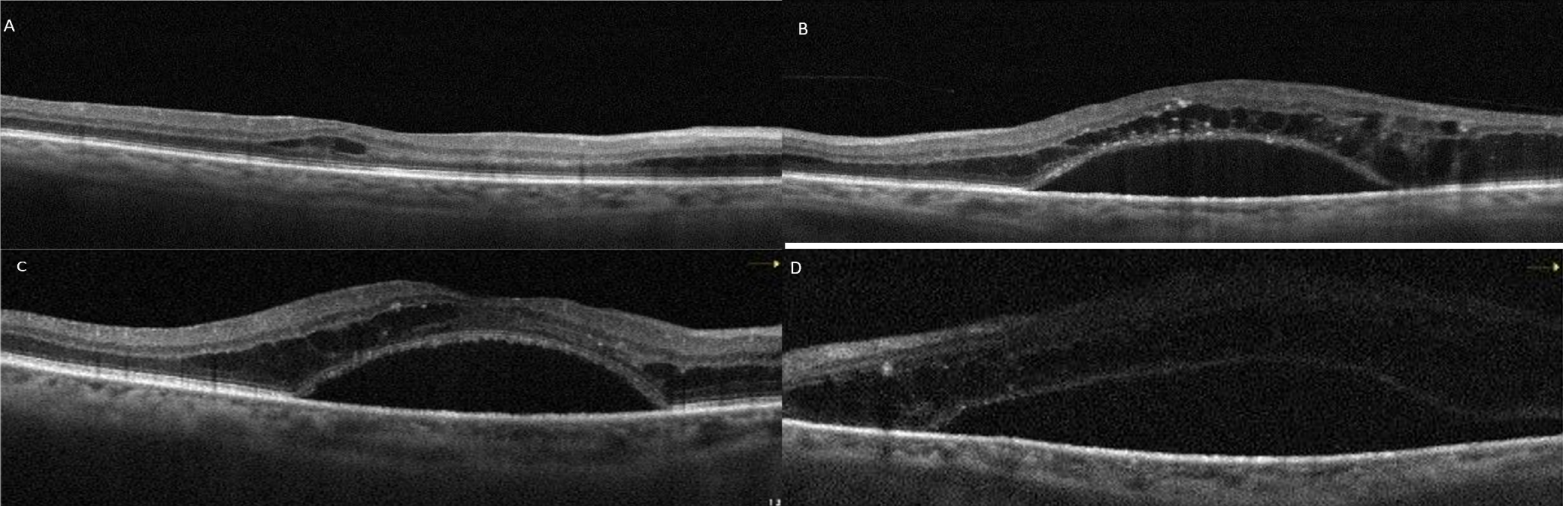
Right (A) and left (B) macular OCT images of case 2 at the first week after left cataract surgery. While mild intraretinal edema was present in the right eye, there was marked subretinal serous fluid accumulation accompanied by intraretinal cysts in the left eye. After 3 monthly intravitreal bevacizumab injections, persistant subretinal fluid is visible in the right (C) and the left (D) eye.

At the patient’s first week follow-up after phacoemulsification surgery, the BCVA of the left eye showed no improvement. Macular OCT images revealed intraretinal and subretinal serous fluid accumulation (Figure 4A and B (Fig. 4)). At that time, laboratory tests showed a hemoglobulin A1c level of 12.1% (reference range: 3.5–6.7%), and a hemoglobin level of 13.6 g/dl (reference range: 12.6–17.4 g/dl). Due to DME, the patient was administered three monthly bilateral intravitreal bevacizumab injections and advised to improve glycemic control. However, the macular edema did not regress; on the contrary, it progressed (Figure 4C and D (Fig. 4)). Subsequently, the patient was lost to follow-up. One year later, ophthalmological examination revealed a BCVA of 20/200 in the right eye and counting fingers at 1 m in the left eye. Slit-lamp examination revealed bilateral pseudophakia, with mild posterior subcapsular opacification in the left eye. No signs of rubeosis iridis were observed. IOPs were within normal limits in both eyes. Fundus examination of the right eye disclosed extensive retinal hemorrhage, hard exudates, microaneurysms, and macular thickening. Visualization of the left fundus was not possible; however, ultrasonographic evaluation indicated findings consistent with intravitreal hemorrhage. Upon further assessment, it was revealed that the patient had been diagnosed with MM approximately one month earlier and had since initiated chemotherapy. At that time, the patient’s hemoglobin level was 11.7 g/dL (reference range: 13.2–16.6 g/dL), and hemoglobin A1c was 6.7% (reference range: 3.5–5.7%). The patient was advised to maintain an upright sitting position, and continue the systemic treatment.

**Figure 5:**
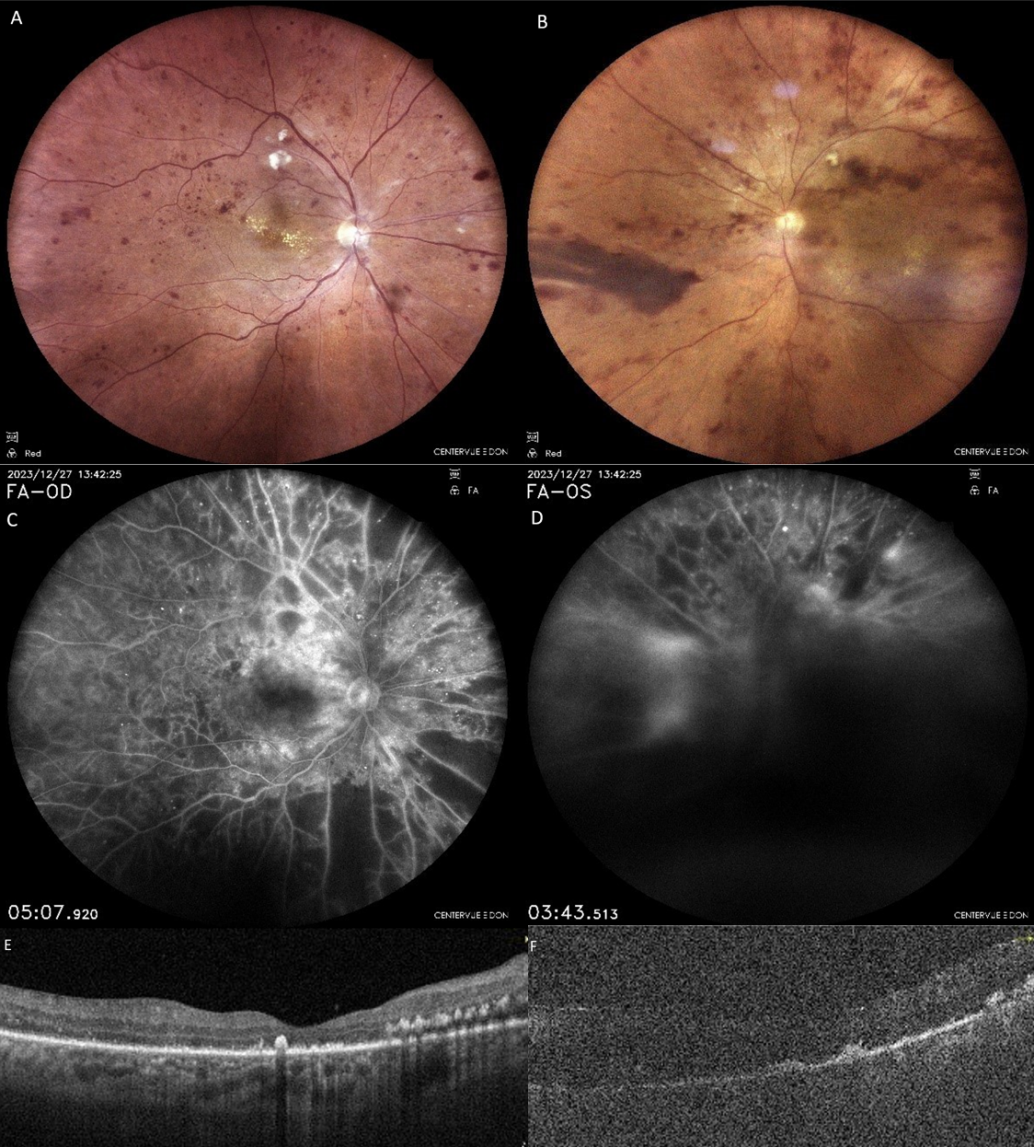
Color fundus (A, B), FFA (C, D) and macular OCT (E, F) images of case 2 at the second month following systemic chemotherapy. Capillary dropouts were noted in the right eye and in visible areas of the left eye in FFA. OCT shows regression of macular edema and disrupted ellipsoid zone on the right eye; the image quality of the left eye is poor, preventing a definitive assessment.

Two months later, partial clearance of vitreous hemorrhage was observed (Figure 5A and B (Fig. 5)). Due to the presence of widespread retinal nonperfusion areas on FFA (Figure 5C and D (Fig. 5)), panretinal laser treatment was initiated. At that time, BCVA was 20/200 in the right eye and counting fingers at 2 m in the left eye. OCT demonstrated regression of macular edema in the right eye; however, hard exudates remained prominent, and disruption of the ellipsoid zone was noted (Figure 5E (Fig. 5)). The left eye remained difficult to visualize, although OCT findings suggested some resolution of the edema (Figure 5F (Fig. 5)). Approximately eight months after the initiation of systemic treatment, the hemorrhages in the right eye showed partial regression. Vitreous hemorrhage in the left eye had significantly cleared.

## Discussion

In this report, the response to intravitreal therapy was insufficient for two diabetic patients who presented with macular edema accompanied by SRD. Subsequent systemic evaluation revealed PM, a condition that may mimic DME. Notably, maculopathy regressed following systemic treatment. PM has been reported in association with a range of systemic disorders. MM is the most severe form of plasma cell dyscrasias, and excessive production of monoclonal immunoglobulin or its fragments by plasma cells causes systemic complications associated with the disease [5]. The clinical course of MM often begins with an asymptomatic monoclonal gammopathy. When it progresses to the symptomatic form, it can eventually lead to organ damage such as renal dysfunction, osteolytic bone lesions, anemia or hypercalcemia [6]. Although fatigue and bone pain are the most common symptoms, ocular involvement has also been reported, even during the early stages of the disease [4], [5], [6], [7], [8], [9], [10], [11], [12], [13], [14], [15]. Such involvement may result from various pathogenic mechanisms, including direct deposition of immunoglobulin light chains in tissues (cornea, conjunctiva, ciliary pigment epithelium, ciliary body, and subretinal pigment epithelium), retinal manifestations of hyperviscosity syndrome, mechanical compression by extramedullary plasmacytomas, metastatic infiltration, and paraneoplastic retinal degeneration [15]. Signs of hyperviscosity retinopathy include retinal vein engorgement, vessel tortuosity, scattered hemorrhages, microaneurysms, optic disc edema, and venous beading, and may closely resemble DR [16]. In the present report, both patients had a history of diabetes mellitus; however, no significant increase in venous tortuosity or vessel engorgement was observed. Instead, microaneurysms and retinal hemorrhages were more prominent in the fundus examination. The presence of intraretinal cysts accompanied by the subretinal fluid initially supported a diagnosis of DME. Nevertheless, a suboptimal response to intravitreal anti-VEGF therapy prompted further investigation for alternative maculopathies. Similarly, Jayadev et al. described a diabetic patient with persistent macular edema refractory to multiple intravitreal anti-VEGF and steroid treatments. Upon systemic evaluation, the patient was ultimately diagnosed with MM [4]. Notably, it has been previously reported that subretinal exudation, which may occur in diabetic maculopathy, could facilitate the accumulation of abnormal immunoglobulins in the subretinal space, particularly in rare cases of presumed PM. This accumulation may enhance the osmotic gradient and contribute to disease progression [17]. PM can present unilaterally and can be misdiagnosed as DME, adult vitelliform dystrophy, or central serous chorioretinopathy. However, unlike these entities, the management of PM focuses on reducing serum immunoglobulin levels through systemic intervetions such as plasmapheresis, chemotherapy, and systemic corticosteroids [1].

## Conclusion

In diabetic patients, OCT alone may not be sufficient for a comprehensive evaluation of the macula. When clinical suspicion arises, a detailed medical history should be obtained. Notably, in patients with bilateral, treatment-resistant SRD, underlying paraproteinemic disorders – such as monoclonal gammopathies and other plasma cell dyscrasias – should also be considered in the differential diagnosis. 

## Abbreviations

- BCVA: best-corrected visual acuity
- DME: diabetic macular edema
- DR: diabetic retinopathy
- FFA: fundus fluorescein angiography
- IOP: intraocular pressure
- MM: multiple myeloma
- OCT: optical coherence tomography
- PM: paraproteinemic maculopathy
- SRD: serous retinal detachment
- VEGF: vascular endothelial growth factor

## Notes

### Authors’ contributions

- Conceptualization: Acan D, Girgin Y, Karahan E
- Data curation: Girgin Y
- Formal analysis: Girgin Y, Acan D
- Funding acquisition: Acan D
- Investigation: Acan D, Girgin Y, Karahan E
- Methodology: Acan D, Girgin Y, Karahan E
- Project administration: Karahan E
- Resources: Girgin Y
- Supervision: Karahan E
- Validation: Acan D, Girgin Y
- Writing – original draft: Acan D
- Writing – review & editing: Acan D, Karahan E

### Preprint 

This manuscript was previously posted to preprints.org [18].

### Data availability 

The datasets generated and/or analyzed during the current study are available from the corresponding author upon reasonable request.

### Informed consent 

Written informed consent was obtained from both patients for publication of this case report and any accompanying images.

### Ethics statement 

All procedures performed in studies involving human participants were in accordance with the ethical standards of the national research committee and with the 1964 Helsinki Declaration.

### Competing interests

The authors declare that they have no competing interests.
